# Health care epidemiology, characteristics, and regional variation of chiropractic care in Switzerland: a descriptive study using insurance claims data

**DOI:** 10.1186/s12913-025-13801-7

**Published:** 2026-02-05

**Authors:** Javier Muñoz Laguna, Lea S. Rohner, Malin Mühlemann, Andri Signorell, Laura C. Rosella, Milo A. Puhan, Cesar A. Hincapié

**Affiliations:** 1 Musculoskeletal Epidemiology Research Group, Epidemiology, Biostatistics and Prevention Institute (EBPI) and University Spine Centre Zurich (UWZH), University of Zurich and Balgrist University Hospital, Zurich, Switzerland; 2 Epidemiology, Biostatistics and Prevention Institute (EBPI), University of Zurich, Zurich, Switzerland; 3 University Spine Centre Zurich (UWZH), Balgrist University Hospital, University of Zurich, Zurich, Switzerland; 4 Helsana Group, Zurich, Switzerland; 5 Dalla Lana School of Public Health, University of Toronto, Toronto, Canada; 6 ICES, Ontario, Canada; 7 Institute for Better Health, Trillium Health Partners, Mississauga, Canada; 8 Temerty Faculty of Medicine, University of Toronto, Toronto, Canada

**Keywords:** Chiropractic, Health care epidemiology, Health care variation, Regional variation, Health administrative data

## Abstract

**Background:**

Musculoskeletal disorders are a leading cause of disability and major driver of health care costs in Switzerland. Since chiropractors are musculoskeletal health care providers in this setting, we aimed to describe the health care epidemiology, characteristics, and regional variation of chiropractic care in Switzerland.

**Method:**

We conducted a retrospective descriptive study using administrative health insurance claims data of all ages from Helsana. Chiropractic care incidence cases were persons with first occurrence of chiropractic care in two study index years, 2018 and 2019. Chiropractic care prevalence cases had any chiropractic care over a 24-month prevalence period. We estimated (i) crude, (ii) sex-, age-, and region- standardised 12-month cumulative incidence, and (iii) 24-month prevalence of chiropractic care per 100,000 population. We characterised four mutually exclusive chiropractic care incidence subgroups based on additional health care use eight weeks before or after the index visit. Regional variation was visualised with choropleth maps.

**Results:**

Among 1,137,904 and 1,196,760 persons with Helsana health insurance in 2018 and 2019, 17,148 (mean age 48.7 [SD, 18.4] years; 9,717 [56.7%] women) and 18,261 (mean age 48.2 [SD, 18.2] years; 10,421 [57.0%] women) persons had a chiropractic care incidence visit, respectively. The crude 12-month chiropractic care incidence was estimated at 1,507 (95% CI, 1,485 to 1,530) and 1,526 (1,504 to 1,548) cases per 100,000 in 2018 and 2019, respectively. Sex-, age-, and region-standardised incidence estimates were comparable to crude estimates. The 24-month prevalence of chiropractic care was estimated at 3,135 (3,105 to 3,166) per 100,000 population. The chiropractic case subgroup with the highest health care use had more comprehensive insurance models with lower deductibles. Incidence of chiropractic care varied across regions with the lowest incidence observed in Ticino and the highest in Espace Mittelland and Zurich.

**Conclusions:**

The incidence and prevalence of chiropractic care utilisation in Switzerland in 2018 and 2019 were markedly low. Chiropractic care subgroups differed in their characteristics, and incidence estimates varied across major regions. Further studies on the predictors and causes of chiropractic care underutilisation are warranted to inform accessible and equitable musculoskeletal and rehabilitation health services in Switzerland.

**Supplementary information:**

The online version contains supplementary material available at 10.1186/s12913-025-13801-7.

## Introduction

Musculoskeletal disorders are the leading cause of years lived with disability worldwide [1]. Despite the advancement of health services knowledge [2, 3], musculoskeletal disorders drive substantial costs [4] and health care spending [5].

In Switzerland, chiropractors provide guideline-recommended care and manage musculoskeletal conditions conservatively [6, 7]. Little is known, however, about chiropractic utilisation in this setting and how it integrates within different care pathways and providers. Characterising the health care epidemiology, utilisation patterns, and regional variation in chiropractic care across Switzerland is essential to inform evidence-based planning and policy development.

Chiropractic care data sources and utilisation estimates vary across the globe [8–11]. In Switzerland, the compulsory (basic) health insurance system covers and routinely collects data on all chiropractic care. Since all residents of Switzerland are required by law to have compulsory health insurance coverage, health insurance administrative data present a valuable information source to gain understanding on the health care epidemiology, characteristics, and regional variation of chiropractic care. Leveraging these data sources may address an evidence gap in Swiss chiropractic and advance knowledge in the areas of musculoskeletal and rehabilitation health services research.

Our overarching aim was to describe the health care epidemiology, characteristics, and regional variation of chiropractic care in Switzerland in 2018 and 2019. Our specific objectives were: (i) to estimate crude, sex-, age-, and region-standardised 12-month cumulative incidence of chiropractic care, (ii) to estimate the crude 24-month prevalence of chiropractic care, (iii) to characterise four mutually exclusive chiropractic care incidence subgroups based on additional health care use eight weeks before or after a chiropractic care index visit date, and (iv) to explore and visualise regional health care variation by estimating incidence of chiropractic care in seven major regions of Switzerland (Lake Geneva region, Espace Mittelland, North-West Switzerland, Zurich region, Eastern Switzer­land, Central Switzerland, and Ticino; ‘major regions’ hereafter). Our descriptive estimand for our measure of occurrence (incidence, prevalence) is further defined in terms of the target population (permanent residents of all ages living in Switzerland between 1 January 2018 and 31 December 2019; alive through this period; with a minimum of one year compulsory health insurance coverage), health care outcome to be summarised (chiropractic care), and standardisation variables (sex, age, and region).

## Methods

### Design

We conducted a retrospective descriptive longitudinal study using administrative Helsana health insurance claims data. We adhered to the RECORD reporting guideline—an extension of the STROBE statement for observational studies with routinely collected health data [12] (sTable 1). Our reporting was also informed by a descriptive epidemiology framework [13]. The study was preregistered in the Open Science Framework (OSF) [14].

### Setting and source population

Health insurance is compulsory in Switzerland. There are more than 50 insurance companies providing health insurance coverage in Switzerland. Helsana is one of the largest national health insurers. At the time our study data were collected, Helsana had approximately 15% market share [15].

The source population was all residents of Switzerland (all ages) insured with Helsana in two study index years—2018 and 2019. To comply with Helsana data sharing policy, employees and persons with less than one year of insurance coverage prior to the index year were excluded from the source population.

### Study population—persons with chiropractic care incidence claims

We conceptualised chiropractic care cases as persons with a chiropractic care incidence claim in 2018 or 2019, with a look-back period of one year for cases in 2018. For each chiropractic care case in the 2018 and 2019 index years, Helsana provided data for one year before, the index year, and two years after the index year. Hence, the overall study period was from 1 January 2017 (time origin for chiropractic care incidence cases in 2018) to 31 December 2021 (end of two-year follow-up period for 2019 cases). One Helsana employee (AS) had access to and queried the Helsana data source to create the dataset for this study.

Chiropractic care incidence was operationalised as first occurrence (index visit date) of service tariff type 324 (chiropractic service group), with billing codes 6001 or 6006 (a chiropractic initial consultation [6001] or differential diagnosis consultation for a new complaint [6006]) (see sTable 2 for details on service tariff codes). We estimated 12-month cumulative incidence per 100,000 population for the periods (i) 1 January 2018 to 31 December 2018, and (ii) 1 January 2019 to 31 December 2019.

### Chiropractic care prevalence

Chiropractic care prevalence was operationalised as the occurrence of any chiropractic care (see sTable 2 for coding). We estimated the 24-month prevalence per 100,000 population for the period 1 January 2018 to 31 December 2019.

### Chiropractic care case subgroups

Among chiropractic care incidence cases, we prespecified four mutually exclusive subgroups based on additional health care use eight weeks before or after the chiropractic index visit date. This eight-week period was prespecified on the basis of clinical judgment to capture common health care pathways and characterise health care use subgroups related to chiropractic care incidence:

- Subgroup 1—Persons who had (i) a visit with a general practitioner, or orthopaedist, or rheumatologist (GP/Ortho/Rheum).
- Subgroup 2—Persons who had (i) a visit with a general practitioner, or orthopaedist, or rheumatologist, and (ii) spine-related imaging (GP/Ortho/Rheum + Imaging).
- Subgroup 3—Persons who had (i) a visit with a general practitioner, or orthopaedist, or rheumatologist, and (ii) spine-related imaging, and (iii) physiotherapy or spine-related corticosteroid injection (GP/Ortho/Rheum + Imaging + PT/Injection).
- Subgroup 4—Persons with no additional health care use as described above, in Subgroups 1 to 3 (No Additional Care).

### Characteristics

We characterised our source population and chiropractic care incidence cases in terms of sex (women and men), mean age, age groups (0 to 17, 18 to 29, 30 to 69, and 70 years or older), number and percentage of deceased in each year, major region, language region (German, French, Italian, and Romansch), health insurance model (standard [free choice], family physician [binding], telemedicine [binding], and telemedicine [nonbinding]), and health insurance deductible (levels 1 [≤500 CHF], 2 [501–1500 CHF], 3 [1501–2500 CHF]). We further described the number of comorbidities in the index year based on pharmaceutical cost groups (none, one, two, and three or more; see sTable 3), and whether the chiropractic index visit was in-hospital.

We characterised unique chiropractic care incidence cases and subgroups based on the above variables. In addition, we described their health care use in the chiropractic index year (outpatient primary, outpatient specialist, outpatient hospital, and inpatient hospital care), and occurrence of spine surgery (during year prior to index year, during index year, and during two-year follow-up after index year). Both health care use and spine surgery variables were based on the presence of any claim-specific billed services.

### Statistical analysis

#### Incidence estimation

The crude 12-month chiropractic care incidence per 100,000 population for the index periods (2018 and 2019) in the Helsana source population was estimated as the number of persons with chiropractic care incidence divided by all insured persons with Helsana—the ‘at-risk’ population for experiencing a new chiropractic visit for a new complaint.

To characterise our health care outcome distribution, we estimated crude 12-month incidence stratified by levels of sex, age and region. To improve and extend our inferences to our target general (permanent resident) population of Switzerland, and under a set of assumptions, we estimated (i) sex-, (ii) age-, and (iii) region-standardised incidence with the direct method [16, 17].

We then estimated the 24-month chiropractic care prevalence by considering unique persons with any chiropractic care in 2018 and 2019.

To report precision around crude incidence and prevalence point estimates, confidence intervals were calculated using the Wilson method [18]. For confidence intervals in standardised incidence estimates, we used the Dobson method [19]. We set the level of type-I error for confidence intervals to 0.05.

#### Subgroup characterisation

We used descriptive statistics—means and confidence intervals for continuous variables and numbers and percentages for categorical variables—to summarise characteristics of the chiropractic care incidence case population.

#### Regional variation

To explore regional variation, we performed stratified analyses and estimated crude incidence by levels of seven major regions. We also estimated region-standardised incidence for 2018 and 2019 and visualised regional variation with choropleth maps. Choropleth maps use colour gradients to represent numerical values across geographical locations, and have been historically utilised to illustrate health care variation [20].

#### Sensitivity and post hoc analyses

For the primary analysis and estimation of 2019 incidence, we considered that the 2018 cases were still part of the at-risk population for experiencing a chiropractic initial consultation or a differential diagnosis consultation for a new complaint. To assess the robustness of the 12-month 2019 incidence estimates, we performed a sensitivity analysis removing from the numerator (i.e., incidence cases) and the denominator (i.e., the at-risk population) (i) all persons with previous chiropractic care incidence in 2018 (most conservative), (ii) persons with less than 180 days between their chiropractic index visit dates in 2018 and 2019, and (iii) persons with less than 90 days between their chiropractic index visit dates in 2018 and 2019 (least conservative). For added transparency and clarity, we characterised persons with chiropractic care incidence in both 2018 and 2019.

To provide further context on the regional variation and supply of chiropractic care, we tabulated crude incidence estimates along with the number of chiropractors in each major region.

Missing data were described using numbers and percentages. All analyses were performed in R [21–24].

## Results

### Incidence

**Fig. 1:**
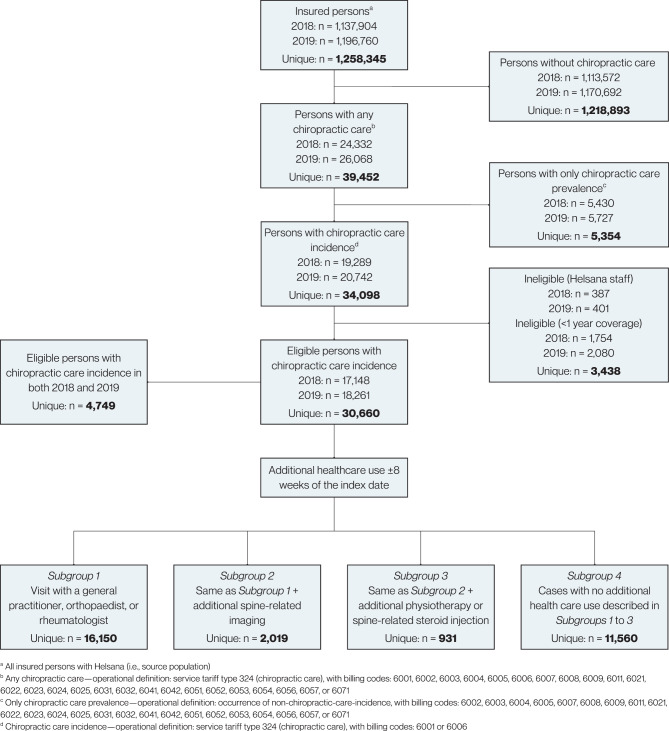
Study population flow and selection

Among 1,137,904 and 1,196,760 persons with Helsana compulsory health insurance coverage in 2018 and 2019, 17,148 (mean age 48.7 [SD, 18.4] years; 9,717 [56.7%] women) and 18,261 (mean age 48.2 [SD, 18.2] years; 10,421 [57.1%] women) persons had 12-month chiropractic care incidence, respectively. The crude 12-month incidence of chiropractic care was estimated at 1,507 (95% CI, 1,485 to 1,530) and 1,526 (1,504 to 1,548) cases per 100,000 population in 2018 and 2019 (Fig. 1; Table 1).

**Table 1:** Characteristics of the source and chiropractic care incidence case population along with the 12-month incidence of chiropractic care per 100,000 population

	Source 2018 *N* = 1,137,904	Cases 2018 *N* = 17,148	Incidence 2018 (95% CI)	Source 2019 *N* = 1,196,760	Cases 2019 *N* = 18,261	Incidence 2019 (95% CI)
Sex – N (%)			*Crude*			*Crude*
Women	588,130 (51.7)	9,717 (56.7)	1,652 (1,620 to 1,685)	616,446 (51.5)	10,421 (57.1)	1,690 (1,659 to 1,723)
Men	549,774 (48.3)	7,431 (43.3)	1,352 (1,321 to 1,383)	580,314 (48.5)	7,839 (42.9)	1,351 (1,321 to 1381)
All sex groups			1,507 (1,485 to 1,530)			1,526 (1,504 to 1,548)
Missing	0 (0)	0 (0)		0 (0)	1 (0)	
			*Sex-standardised*^*a*^ 1,503 (1,481 to 1,526)			*Sex-standardised*^*a*^ 1,522 (1,500 to 1,544)
Age (years) – mean (SD)	43.8 (24.6)	48.7 (18.4)		43.3 (24.5)	48.2 (18.2)	
Age group – N (%)			*Crude*			*Crude*
0 to 17 years	204,070 (17.9)	788 (4.6)	386 (360 to 414)	219,993 (18.4)	830 (4.5)	377 (353 to 404)
18 to 29 years	160,803 (14.1)	2,048 (11.9)	1,274 (1,220 to 1,330)	168,110 (14.0)	2,272 (12.4)	1,351 (1,297 to 1,408)
30 to 69 years	567,167 (49.8)	11,755 (68.6)	2,073 (2,036 to 2,110)	600,472 (50.2)	12,574 (68.9)	2,094 (2,058 to 2,131)
70 years or older	205,864 (18.1)	2,557 (14.9)	1,242 (1,195 to 1,291)	208,185 (17.4)	2,584 (14.2)	1,241 (1,195 to 1,290)
All age groups			1,507 (1,485 to 1,530)			1,526 (1,504 to 1,548)
Missing	0 (0)	5 (0)		0 (0)	1 (0)	
			*Age-standardised*^*a*^ 1,544 (1,520 to 1,567)			*Age-standardised*^*a*^ 1,565 (1,542 to 1,588)
Major region – N (%)			*Crude*			*Crude*
Lake Geneva region	188,383 (16.6)	2,539 (14.8)	1,248 (1,297 to 1,401)	201,709 (16.9)	2,823 (15.5)	1,400 (1349 to 1451)
Espace Mittelland	228,634 (20.1)	4,332 (25.3)	1,895 (1,840 to 1,951)	243,284 (20.3)	4,518 (24.7)	1,857 (1,804 to 1,911)
Northwestern Switzerland	162,307 (14.3)	1,816 (10.6)	1,119 (1,069 to 1,171)	173,953 (14.5)	1,917 (10.5)	1,102 (1,054 to 1,152)
Zurich	273,414 (24.0)	4,741 (27.6)	1,734 (1,686 to 1,784)	285,189 (23.8)	5,245 (28.7)	1,839 (1,790 to 1,889)
Eastern Switzerland	128,973 (11.3)	1,894 (11.0)	1,469 (1,404 to 1,536)	133,821 (11.2)	1,856 (10.2)	1,387 (1,326 to 1,451)
Central Switzerland	88,025 (7.7)	1,459 (8.5)	1,657 (1,575 to 1,744)	89,361 (7.5)	1,526 (8.4)	1,708 (1,625 to 1,795)
Ticino	68,147 (6.0)	362 (2.1)	531 (479 to 589)	69,434 (5.8)	375 (2.1)	540 (488 to 597)
All region groups			1,507 (1,484 to 1,529)			1,526 (1,504 to 1,548)
Missing	21 (0)	5 (0)		9 (0)	1 (0)	
			*Region-standardised*^*a*^ 1,518 (1,495 to 1,541)			*Region-standardised*^*a*^ 1,530 (1,508 to 1,553)
Deceased – N (%)	12,075 (1.1)			12,118 (1.0)		
Deceased year – N (%)						
2017		0 (0)			-	
2018		1 (0)			0 (0)	
2019		71 (0.4)			0 (0)	
2020		89 (0.5)			74 (0.4)	
2021		-			92 (0.5)	
Missing	0 (0)	0 (0)		0 (0)	0 (0)	
Area of residence by language – N (%)						
German	838,928 (73.7)	13,686 (79.8)		878,169 (73.4)	14,345 (78.6)	
French	225,985 (19.9)	3,067 (17.9)		244,229 (20.4)	3,516 (19.3)	
Italian	70,472 (6.2)	373 (2.2)		71,862 (6.0)	383 (2.1)	
Romansch	2,498 (0.2)	17 (0.1)		2,491 (0.2)	16 (0.1)	
Missing	21 (0)	5 (0)		9 (0)	1 (0)	
Health insurance model – N (%)						
Standard (free choice) model	366,936 (32.2)	8,620 (50.3)		345,144 (28.8)	8,469 (46.4)	
Family physician model^b^	518,527 (45.6)	4,642 (27.1)		574,330 (48.0)	5,432 (29.7)	
Telemedicine model (binding)	167,063 (14.7)	2,332 (13.6)		197,452 (16.5)	2,788 (15.3)	
Telemedicine model (nonbinding)	85,378 (7.5)	1,554 (9.1)		79,834 (6.7)	1,572 (8.6)	
Missing	0 (0)	0 (0)		0 (0)	0 (0)	
Health insurance deductible – N (%)						
Level 1, ≤500 CHF	817,132 (71.8)	10,305 (60.1)		836,559 (69.9)	10,835 (59.3)	
Level 2, 501–1500 CHF	93,250 (8.2)	3,939 (23.0)		90,107 (7.5)	4,020 (22.0)	
Level 3, 1501–2500 CHF	227,522 (20.0)	2904 (16.9)		270,094 (22.6)	3,405 (18.6)	
Missing	0 (0)	0 (0)		0 (0)	1 (0)	
Comorbidities in index year – N (%)						
None		12,331 (71.9)			13,202 (72.3)	
One		3,256 (19.0)			3,495 (19.1)	
Two		857 (5.0)			887 (4.9)	
Three or more		210 (1.2)			239 (1.3)	
Missing		494 (2.9)			438 (2.4)	
In-hospital index visit – N (%)		316 (1.8)			322 (1.8)	
Missing		0 (0)			0 (0)	

^a^ Standardisation performed using general (permanent resident) population of Switzerland as reference [16, 17]; Reference day: 31 December; Data source: Population and Households Statistics STATPOP

^b^ Combines binding and nonbinding insurance models

### Incidence by sex and age

The crude 12-month incidence of chiropractic among women was estimated at 1,652 (1,620 to 1,685) and 1,690 (1,659 to 1,723) per 100,000 population in 2018 and 2019, respectively. Among men, incidence estimates were 1,352 (1,321 to 1,383) and 1,351 (1,321 to 1381) per 100,000 population in 2018 and 2019. Sex-standardised incidence estimates were 1,503 (1,481 to 1,526) and 1,522 (1,500 to 1,544) per 100,000 population.

The lowest 12-month crude incidence estimates were found in the age group of 0 to 17 years at 386 (360 to 414) and 377 (353 to 404) per 100,000 population in 2018 and 2019. The highest crude incidence estimates were in the age group of 30 to 69 years with estimates of 2,073 (2,036 to 2,110) and 2,094 (2,058 to 2,131) per 100,000 population. Age-standardised incidence was 1,544 (1,520 to 1,567) and 1,565 (1,542 to 1,588) in 2018 and 2019 (Table 1).

### Prevalence

Among 1,258,345 unique persons with Helsana health insurance coverage in 2018 and 2019, 39,452 were identified as chiropractic care prevalence cases for the 24-month prevalence period. Hence, the crude prevalence of chiropractic care use was estimated at 3,135 (95% CI, 3,105 to 3,166) per 100,000 population.

### Chiropractic care case subgroups

Chiropractic care case subgroups varied in their characteristics.

Chiropractic care cases who had additional health care use within eight weeks before or after their index date were more often women and older persons. Cases with any health care use within eight weeks of the index date were also more often under standard insurance models with lower health insurance deductibles (Table 2).

**Table 2:** Characteristics of chiropractic care incidence subgroups

		**Subgroups based on health care use before or after 8 weeks of chiropractic care incidence index date**^**a**^			
	Total unique **N = 30,660**^**b**^	Subgroup 1—GP/Ortho/Rheum N = 16,150	Subgroup 2—GP/Ortho/Rheum + Imaging N = 2,019	Subgroup 3—GP/Ortho/Rheum + Imaging + PT/Injection N = 931	Subgroup 4—No Additional Care N = 11,560
Sex – N (%)					
Women	17,413 (56.8)	9,643 (59.7)	1,111 (55.0)	558 (59.9)	6,101 (52.8)
Men	13,246 (43.2)	6,507 (40.3)	907 (44.9)	373 (40.1)	5,459 (47.2)
Missing	1 (0)	0 (0)	1 (0)	0 (0)	0 (0)
Age (years)					
Mean (SD)	48.1 (18.4)	50.6 (18.0)	54.9 (17.1)	54.5 (17.4)	42.9 (17.8)
Age group – N (%)					
0 to 17 years	1,434 (4.7)	400 (2.5)	19 (0.9)	14 (1.5)	1,001 (8.7)
18 to 29 years	3,894 (12.7)	1,942 (12.0)	148 (7.3)	63 (6.8)	1,741 (15.1)
30 to 69 years	20,942 (68.3)	11,016 (68.2)	1,373 (68.0)	628 (67.5)	7,925 (68.6)
70 years or older	4,389 (14.3)	2,792 (17.3)	478 (23.7)	226 (24.3)	893 (7.7)
Missing	1 (0)	0 (0)	1 (0)	0 (0)	0 (0)
Major region – N (%)					
Lake Geneva region	4,523 (14.8)	2,094 (13.0)	219 (10.8)	93 (10.0)	2,117 (18.3)
Espace Mittelland	7,581 (24.7)	4,008 (24.8)	425 (21.1)	191 (20.5)	2,957 (25.6)
Northwestern Switzerland	3,273 (10.7)	1,667 (10.3)	251 (12.4)	96 (10.3)	1,259 (10.9)
Zurich	8,683 (28.3)	4,683 (29.0)	601 (29.8)	342 (36.7)	3,057 (26.4)
Eastern Switzerland	3,278 (10.7)	1,821 (11.3)	265 (13.1)	113 (12.1)	1,079 (9.3)
Central Switzerland	2,625 (8.6)	1,515 (9.4)	219 (10.8)	74 (7.9)	817 (7.1)
Ticino	691 (2.3)	360 (2.2)	38 (1.9)	22 (2.4)	271 (2.3)
Missing	6 (0)	2 (0)	1 (0)	0 (0)	3 (0)
Deceased year – N (%)					
2018	1 (0)	0 (0)	0 (0)	0 (0)	1 (0)
2019	71 (0.2)	44 (0.3)	9 (0.4)	4 (0.4)	14 (0.1)
2020	146 (0.5)	91 (0.6)	20 (1.0)	5 (0.5)	30 (0.3)
2021	159 (0.5)	99 (0.6)	20 (1.0)	9 (1.0)	31 (0.3)
Area of residence by language – N (%)					
German	24,315 (79.3)	13,225 (81.9)	1,694 (83.9)	793 (85.2)	8,603 (74.4)
French	5,602 (18.3)	2,539 (15.7)	282 (14.0)	115 (12.4)	2,666 (23.1)
Italian	709 (2.3)	371 (2.3)	38 (1.9)	22 (2.4)	278 (2.4)
Romansch	28 (0.1)	13 (0.1)	4 (0.2)	1 (0)	10 (0.1)
Missing	6 (0)	2 (0)	1 (0.0)	0 (0)	3 (0)
Health insurance model – N (%)					
Standard (free choice) model	14,575 (47.5)	7,850 (48.6)	996 (49.3)	484 (52.0)	5,245 (45.4)
Family physician model (binding)	8,882 (29.0)	5,195 (32.2)	679 (33.6)	293 (31.5)	2,715 (23.5)
Telemedicine model (binding)	4,512 (14.7)	1,863 (11.5)	205 (10.2)	86 (9.2)	2,358 (20.4)
Telemedicine model (nonbinding)	2,691 (8.8)	1242 (7.7)	139 (6.9)	68 (7.3)	1,242 (10.7)
Missing	0 (0)	0 (0)	0 (0)	0 (0)	0 (0)
Health insurance deductible – N (%)					
Level 1, ≤500 CHF	18,192 (59.3)	10,424 (64.5)	1,336 (66.2)	667 (71.6)	5,765 (49.9)
Level 2, 501–1500 CHF	6,876 (22.4)	3,612 (22.4)	484 (24.0)	192 (20.6)	2,588 (22.4)
Level 3, 1501–2500 CHF	5,591 (18.2)	2,114 (13.1)	198 (9.8)	72 (7.7)	3207 (27.7)
Missing	1 (0.0)	0 (0)	1 (0)	0 (0)	0 (0)
Comorbidities in index year – N (%)					
None	22,162 (72.3)	10,846 (67.2)	1,217 (60.3)	545 (58.5)	9,554 (82.6)
One	5,768 (18.8)	3,675 (22.8)	550 (27.2)	247 (26.5)	1,296 (11.2)
Two	1,484 (4.8)	957 (5.9)	156 (7.7)	95 (10.2)	276 (2.4)
Three or more	378 (1.2)	256 (1.6)	43 (2.1)	24 (2.6)	55 (0.5)
Missing	868 (2.8)	416 (2.6)	53 (2.6)	20 (2.1)	379 (3.3)
In-hospital index visit – N (%)	559 (1.8)	259 (1.6)	51 (2.5)	51 (5.5)	198 (1.7)

^a^ Four mutually exclusive subgroups based on additional health care use eight weeks before or after the chiropractic index visit date: Subgroup 1—GP/Ortho/Rheum: Persons who had (i) a visit with a general practitioner, orthopaedist, or rheumatologist; Subgroup 2—GP/Ortho/Rheum + Imaging: Persons who had (i) a visit with a general practitioner, orthopaedist, or rheumatologist, and (ii) spine-related imaging; Subgroup 3—GP/Ortho/Rheum + Imaging + PT/Injection: Persons who had (i) a visit with a general practitioner, orthopaedist, or rheumatologist, and (ii) spine-related imaging, and (iii) physiotherapy or spine-related steroid injection; Subgroup 4—No Additional Care: Persons with no additional health care use described in Subgroups 1 to 3

^b^ Characteristics presented at time of first index year for 4,749 cases that had an eligible index date in both 2018 and 2019

The subgroup of cases with the highest health care use within eight weeks of the index date tended to have more comorbidities and was more likely to receive in-hospital treatment for their chiropractic index visit. Overall, these persons had the highest utilisation across all outcomes in the index year—outpatient primary care, outpatient specialist care, outpatient hospital care, and in-patient hospital care. In addition, they were more likely to receive spine surgery in the index and two-year follow-up (Table 3).

**Table 3:** Healthcare use of chiropractic care incidence subgroups

	**Total unique N = 30,660**^**b**^	**Subgroups based on health care use before or after 8 weeks of chiropractic care incidence index date**^**a**^			
		Subgroup 1—GP/Ortho/Rheum N = 16,150	Subgroup 2—GP/Ortho/Rheum + Imaging N = 2,019	Subgroup 3—GP/Ortho/Rheum + Imaging + PT/Injection N = 931	Subgroup 4—No Additional Care N = 11,560
Health care use in index year – N (%)^c^					
Outpatient primary care	23,653 (77.1)	14,497 (89.8)	1,803 (89.3)	843 (90.5)	6,510 (56.3)
Outpatient specialist care	24,464 (79.8)	13,875 (85.9)	1,869 (92.6)	887 (95.3)	7,833 (67.8)
Outpatient hospital care	16,548 (54.0)	9,495 (58.8)	1,453 (72.0)	772 (82.9)	4,828 (41.8)
Inpatient hospital care	4,118 (13.4)	2,435 (15.1)	411 (20.4)	263 (28.2)	1,009 (8.7)
Spine surgery – N (%)^c^					
During year prior to index year	216 (0.7)	117 (0.7)	26 (1.3)	29 (3.1)	44 (0.4)
During index year	443 (1.4)	192 (1.2)	91 (4.5)	84 (9.0)	76 (0.7)
During 2-year follow-up after index year	539 (1.8)	277 (1.7)	69 (3.4)	67 (7.2)	126 (1.1)

^a^ Four mutually exclusive subgroups based on additional health care use eight weeks before or after the chiropractic index visit date: Subgroup 1—GP/Ortho/Rheum: Persons who had (i) a visit with a general practitioner, orthopaedist, or rheumatologist; Subgroup 2—GP/Ortho/Rheum + Imaging: Persons who had (i) a visit with a general practitioner, orthopaedist, or rheumatologist, and (ii) spine-related imaging; Subgroup 3—GP/Ortho/Rheum + Imaging + PT/Injection: Persons who had (i) a visit with a general practitioner, orthopaedist, or rheumatologist, and (ii) spine-related imaging, and (iii) physiotherapy or spine-related steroid injection; Subgroup 4—No Additional Care: Persons with no additional health care use described in Subgroups 1 to 3

^b^ Characteristics presented at time of first index year for 4,749 cases that had an eligible index date in both 2018 and 2019

^c^ Based on compulsory health insurance billed services

### Regional variation

Incidence of chiropractic care varied across major regions.

In 2018, the highest incidence of chiropractic care use was estimated in the region of Espace Mittelland at 1,895 (1,840 to 1,951) cases per 100,000 population. This region was followed by Zurich at 1,734 (1,686 to 1,784), and Central Switzerland at 1,657 (1,575 to 1,744) cases per 100,000 population. The lowest incidence of chiropractic care was observed in the region of Ticino at 531 (479 to 589) cases per 100,000 population.

**Fig. 2:**
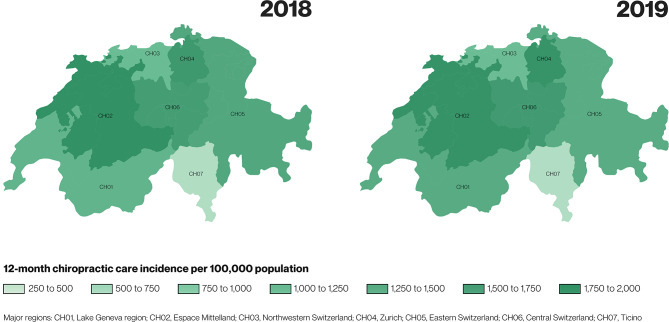
Regional variation in 12-month chiropractic care incidence per 100,000 population in 2018 and 2019

In 2019, the highest incidence of chiropractic care use was estimated in the region of Espace Mittelland at 1,857 (1,804 to 1,911), followed by Zurich at 1,839 (1,790 to 1,889) and Central Switzerland at 1,708 (1,625 to 1,795) per 100,000 population. The lowest incidence was observed in Ticino at 540 (488 to 597), followed by Northwestern Switzerland at 1,102 (1,054 to 1,152) cases per 100,000 population (Fig. 2).

### Sensitivity and post hoc analyses

We re-estimated crude 12-month incidence for the year 2019 after identifying and removing from the numerator and denominator: (i) 4,749 persons with a previous chiropractic incidence visit in 2018, (ii) 473 persons with a previous incidence visit in 2018 and ≤ 180 days between their index visits in 2018 and 2019, and (iii) 99 persons with a previous incidence visit in 2018 and ≤ 90 days between their index visits in 2018 and 2019. Incidence estimates were (i) 1,134 (1,115 to 1,153), (ii) 1,487 (1,465 to 1,509), and (iii) 1,518 (1,496 to 1,540). Persons with chiropractic care incidence in both 2018 and 2019 varied in their characteristics (sTable 4).

We tabulated crude incidence estimates along with the number of chiropractors in each major region of Switzerland. Overall, regional incidence estimates of chiropractic care were not consistent with regional chiropractic supply in 2018 and 2019 (sTable 5).

## Discussion

### Major findings

Among a source population of over a million insured persons in Switzerland, less than two percent were identified as 12-month chiropractic care incidence cases in 2018 and 2019. Just over three percent were identified as chiropractic care prevalence cases over a 24-month prevalence period. Chiropractic care subgroups with more health care use within eight weeks before or after the chiropractic care incidence index visit tended to have more comprehensive insurance models with lower deductibles. They also had on average, more comorbidities, and underwent more spine surgeries during the index year and the two-year follow-up period. We found considerable regional variation in the incidence of chiropractic care.

### Importance and implications

Our findings address an evidence gap in Swiss musculoskeletal and rehabilitation health services and are informative for health care workforce policy in Switzerland. The low 12-month incidence of chiropractic care suggests that most people living with musculoskeletal conditions may be referred or opt to seek care from their primary care physicians [25], or other health care providers [10, 26]. Care seeking behaviour may be influenced by insurance models that require initial contact and evaluation by family physicians. Considering that one in every five primary care visits has a musculoskeletal complaint as a reason for encounter [25], and that burnout among primary care physicians is on the rise [27], further understanding of the potential supportive role that chiropractors could play in managing musculoskeletal disorders is warranted. Furthermore, since underutilisation often coexists with overutilisation of health services [28], better understanding of potential competitive tensions between high- and low-cost interventions for musculoskeletal disorders may be helpful. Health economic evaluations may also help shed light on the cost effectiveness of common musculoskeletal interventions in the Swiss health care setting.

It should be noted that a considerable proportion of people living with musculoskeletal conditions may not seek any care [29]. Yet, our subgroup findings suggest that people with less comprehensive health insurance models and higher deductibles may be experiencing barriers to access care. To improve patient outcomes, policymakers may consider investigating better integration of chiropractic services within primary care to remove potential barriers and foster equitable access to evidence-based, first-line treatments for musculoskeletal complaints [30, 31].

The characteristics of different chiropractic care incidence case subgroups provide a glimpse of patterns of health care use adjacent to chiropractic care [32]. The considerable variation in chiropractic care use across Swiss regions was not consistent with the regional distribution and supply of chiropractors [33]. Further investigation into the causes of this regional variation, including patient preferences, physician referral patterns, cultural attitudes, and accessibility barriers, is warranted to identify potential unmet needs or underutilisation [34, 35].

Policy implications of our findings may include an emphasis on programs that target (i) family physician knowledge of chiropractic care, (ii) the development of connections between family physicians and chiropractors in the community, and (iii) the reduction of logistical challenges in family physician referral processes [36]. These programs deserve thoughtful evaluation and may be an area of interest for future research.

### Context

To our knowledge, this is the first study to describe the health care epidemiology, characteristics, and regional variation of chiropractic care in Switzerland. Our findings should be interpreted alongside prevalence estimates for musculoskeletal disorders in our setting (2018: 37,200 [95% UI, 35,500 to 38,900]; 2019: 37,100 [95% UI, 35,300 to 38,800] per 100,000 population) [37]. Our incidence estimates contrast with the findings of a scoping review of chiropractic utilisation [8], which reported that the global median 12-month utilisation of chiropractic services was 9,100 (interquartile range [IQR]: 6,700 to 13,100) per 100,000 population. Relative to other countries such as Australia, United States, and Canada, our utilisation estimates were markedly low [8]. It should be noted, however, that (i) there is marked variation in chiropractic utilisation around the globe and that (ii) chiropractic utilisation case definitions differ, which compromises between-study comparability. Consistent with previous studies, our chiropractic care incidence cases were more often female and middle aged [8]. Our considerable regional variation in incidence of chiropractic care highlight the need for small-area analyses (e.g., at the cantonal level) and investigations of supply-sensitive chiropractic care [38, 39].

### Limitations

Our study has limitations. First, our chiropractic case definition is derived from health care utilisation connected to compulsory health insurance in 2018 and 2019 and may be susceptible to varying coding practices and misclassification. For instance, the percentage of insured persons paying out of pocket for chiropractic care services during our study period cannot be estimated. Second, we lacked information on diagnostic codes, reasons for seeking care, referrals, patient-reported outcomes, and socio-economic information in our source and study populations. Third, although our source population may be comparable to the general population in terms of sex and younger age group distributions, it differed in other characteristics (sTable 6). Differences in regional distribution and other unmeasured characteristics may limit the external validity of our findings.

### Strengths

There are also strengths. We pre-registered our protocol and prespecified methodological choices. In addition, we used claims data from a major Swiss health insurance and stratified chiropractic incidence estimates by sex, age, and region to gain a more comprehensive understanding of the health care epidemiology of chiropractic care. Our descriptive characterisation of chiropractic subgroups and our exploration of regional variation are also informative for future research questions on the health care costs associated chiropractic care in Switzerland.

## Conclusion

The incidence and prevalence of chiropractic care utilisation in Switzerland in 2018 and 2019 were markedly low. Chiropractic care subgroups differed in their characteristics, and incidence estimates varied considerably across major regions. Further studies on the predictors and causes of chiropractic care underutilisation are warranted to inform musculoskeletal and rehabilitation health services workforce policy in Switzerland, and ultimately to enhance access, delivery, and outcomes for patients.

## Electronic supplementary material

Below is the link to the electronic supplementary material.

**Supplemental material 1:** Supplemental Tables (sTables) 1 to 6.

## Data Availability

The data that support the findings of this study are subject to third party restrictions. The code underpinning the statistical analysis is available from the corresponding or first author upon reasonable request.

## Abbreviations

CHF: Swiss francs
CI: Confidence interval
HRA: Human Research Act
RECORD: REporting of studies Conducted using Observational Routinely-collected Data
SD: Standard deviation
STROBE: STrengthening the Reporting of OBservational studies in Epidemiology
UI: Uncertainty interval
