# Supplementary material for: Health care epidemiology, characteristics, and regional variation of chiropractic care in Switzerland: a descriptive study using insurance claims data
Source: BMC Health Serv Res. 2026 Feb 5;26:277. doi: 10.1186/s12913-025-13801-7 (PMC12922438; doi:10.1186/s12913-025-13801-7)
Supplement: Supplementary file 1 — Supplemental material 1: Supplemental Tables (sTables) 1 to 6. [file 12913_2025_13801_MOESM1_ESM.docx]

**Supplemental material**

Muñoz Laguna J, Rohner LS, Mühlemann M, Signorell A, Rosella LC, Puhan MA, Hincapié CA. Health care epidemiology, characteristics, and regional variation of chiropractic care in Switzerland: a descriptive study using insurance claims data. BMC Health Serv Res. 2026

**Contents**

**sTable 1.** RECORD statement 2

**sTable 2.** Tariff codes identifying chiropractic care in 2018 and 2019^a^ 9

**sTable 3.** Pharmaceutical cost groups (PCG) 12

**sTable 4.** Characteristics of incidence cases in both 2018 and 2019 13

**sTable 5.** Crude incidence of chiropractic care and number of chiropractors (supply) in 2018 and 2019 14

**sTable 6.** Characteristics of the general population of Switzerland and the Helsana source population 15

# **sTable 1.** RECORD statement

|  | **Item No.** | **STROBE items** | **Location in manuscript where items are reported** | **RECORD items** | **Location in manuscript where items are reported** |
| --- | --- | --- | --- | --- | --- |
| **Title and abstract** | | | | | |
|  | 1 | (a) Indicate the study’s design with a commonly used term in the title or the abstract (b) Provide in the abstract an informative and balanced summary of what was done and what was found |  | RECORD 1.1: The type of data used should be specified in the title or abstract. When possible, the name of the databases used should be included.  RECORD 1.2: If applicable, the geographic region and timeframe within which the study took place should be reported in the title or abstract.  RECORD 1.3: If linkage between databases was conducted for the study, this should be clearly stated in the title or abstract. | ✓ |
| **Introduction** | | | | | |
| **Background rationale** | 2 | Explain the scientific background and rationale for the investigation being reported |  |  | ✓ |
| **Objectives** | 3 | State specific objectives, including any prespecified hypotheses |  |  | ✓ |
| **Methods** | | | | | |
| **Study Design** | 4 | Present key elements of study design early in the paper |  |  | ✓ |
| **Setting** | 5 | Describe the setting, locations, and relevant dates, including periods of recruitment, exposure, follow-up, and data collection |  |  | ✓ |
| **Participants** | 6 | *(a) Cohort study* - Give the eligibility criteria, and the sources and methods of selection of participants. Describe methods of follow-up  *Case-control study* - Give the eligibility criteria, and the sources and methods of case ascertainment and control selection. Give the rationale for the choice of cases and controls  *Cross-sectional study* - Give the eligibility criteria, and the sources and methods of selection of participants  *(b) Cohort study* - For matched studies, give matching criteria and number of exposed and unexposed  *Case-control study* - For matched studies, give matching criteria and the number of controls per case |  | RECORD 6.1: The methods of study population selection (such as codes or algorithms used to identify subjects) should be listed in detail. If this is not possible, an explanation should be provided.  RECORD 6.2: Any validation studies of the codes or algorithms used to select the population should be referenced. If validation was conducted for this study and not published elsewhere, detailed methods and results should be provided.  RECORD 6.3: If the study involved linkage of databases, consider use of a flow diagram or other graphical display to demonstrate the data linkage process, including the number of individuals with linked data at each stage. | ✓ |
| **Variables** | 7 | Clearly define all outcomes, exposures, predictors, potential confounders, and effect modifiers. Give diagnostic criteria, if applicable. |  | RECORD 7.1: A complete list of codes and algorithms used to classify exposures, outcomes, confounders, and effect modifiers should be provided. If these cannot be reported, an explanation should be provided. | ✓ |
| **Data sources/ measurement** | 8 | For each variable of interest, give sources of data and details of methods of assessment (measurement).  Describe comparability of assessment methods if there is more than one group |  |  | ✓ |
| **Bias** | 9 | Describe any efforts to address potential sources of bias |  |  | ✓ |
| **Study size** | 10 | Explain how the study size was arrived at |  |  | ✓ |
| **Quantitative variables** | 11 | Explain how quantitative variables were handled in the analyses. If applicable, describe which groupings were chosen, and why |  |  | ✓ |
| **Statistical methods** | 12 | (a) Describe all statistical methods, including those used to control for confounding  (b) Describe any methods used to examine subgroups and interactions  (c) Explain how missing data were addressed  (d) *Cohort study* - If applicable, explain how loss to follow-up was addressed  *Case-control study* - If applicable, explain how matching of cases and controls was addressed  *Cross-sectional study* - If applicable, describe analytical methods taking account of sampling strategy  (e) Describe any sensitivity analyses |  |  | ✓ |
| **Data access and cleaning methods** |  | .. |  | RECORD 12.1: Authors should describe the extent to which the investigators had access to the database population used to create the study population.  RECORD 12.2: Authors should provide information on the data cleaning methods used in the study. | ✓ |
| **Linkage** |  | .. |  | RECORD 12.3: State whether the study included person-level, institutional-level, or other data linkage across two or more databases. The methods of linkage and methods of linkage quality evaluation should be provided. | NA |
| **Results** | | | | | |
| **Participants** | 13 | (a) Report the numbers of individuals at each stage of the study (*e.g.*, numbers potentially eligible, examined for eligibility, confirmed eligible, included in the study, completing follow-up, and analysed)  (b) Give reasons for non-participation at each stage.  (c) Consider use of a flow diagram |  | RECORD 13.1: Describe in detail the selection of the persons included in the study (*i.e.,* study population selection) including filtering based on data quality, data availability and linkage. The selection of included persons can be described in the text and/or by means of the study flow diagram. | ✓ |
| **Descriptive data** | 14 | (a) Give characteristics of study participants (*e.g.*, demographic, clinical, social) and information on exposures and potential confounders  (b) Indicate the number of participants with missing data for each variable of interest  (c) *Cohort study* - summarise follow-up time (*e.g.*, average and total amount) |  |  | ✓ |
| **Outcome data** | 15 | *Cohort study* - Report numbers of outcome events or summary measures over time  *Case-control study* - Report numbers in each exposure category, or summary measures of exposure  *Cross-sectional study* - Report numbers of outcome events or summary measures |  |  | ✓ |
| **Main results** | 16 | (a) Give unadjusted estimates and, if applicable, confounder-adjusted estimates and their precision (e.g., 95% confidence interval). Make clear which confounders were adjusted for and why they were included  (b) Report category boundaries when continuous variables were categorized  (c) If relevant, consider translating estimates of relative risk into absolute risk for a meaningful time period |  |  | ✓ |
| **Other analyses** | 17 | Report other analyses done—e.g., analyses of subgroups and interactions, and sensitivity analyses |  |  | ✓ |
| **Discussion** | | | | | |
| **Key results** | 18 | Summarise key results with reference to study objectives |  |  | ✓ |
| **Limitations** | 19 | Discuss limitations of the study, taking into account sources of potential bias or imprecision. Discuss both direction and magnitude of any potential bias |  | RECORD 19.1: Discuss the implications of using data that were not created or collected to answer the specific research question(s). Include discussion of misclassification bias, unmeasured confounding, missing data, and changing eligibility over time, as they pertain to the study being reported. | ✓ |
| **Interpretation** | 20 | Give a cautious overall interpretation of results considering objectives, limitations, multiplicity of analyses, results from similar studies, and other relevant evidence |  |  | ✓ |
| **Generalisability** | 21 | Discuss the generalisability (external validity) of the study results |  |  | ✓ |
| **Other Information** | | | | | |
| **Funding** | 22 | Give the source of funding and the role of the funders for the present study and, if applicable, for the original study on which the present article is based |  |  | ✓ |
| **Accessibility of protocol, raw data, and programming code** |  | .. |  | RECORD 22.1: Authors should provide information on how to access any supplemental information such as the study protocol, raw data, or programming code. | ✓ |

*Reference: Benchimol EI, Smeeth L, Guttmann A, Harron K, Moher D, Petersen I, Sørensen HT, von Elm E, Langan SM, the RECORD Working Committee.  The REporting of studies Conducted using Observational Routinely-collected health Data (RECORD) Statement.  *PLoS Medicine* 2015; in press.

*Checklist is protected under Creative Commons Attribution ([CC BY](http://creativecommons.org/licenses/by/4.0/)) license.

# **sTable 2.** Tariff codes identifying chiropractic care in 2018 and 2019^a^

| **Tariff code** | **Meaning^b^** | **Interpretation^b^** |
| --- | --- | --- |
| **01** | Consultations and clinical examinations | Included in the consultation fee are: (i) medical history taking, (ii) general clinical clarification, (iii) indication, education and consultation of the patient, and (iv) diagnostic and simple therapeutic measures for which the tariff does not provide for special compensation. |
| **6001** | Chiropractic initial consultation | A new chiropractic initial consultation. |
| **6002** | Each additional consultation (up to 15 min) | This includes, individually or in combination, among other things:  - Assessment of the subjective and objective course of treatment  - Orthopaedic and neurological control  - Assessment of spinal statics and dynamics  - Movement and work physiological as well as general dietary consultation |
| **6004** | Telephone consultation | Telephone consultation is understood to mean only professional consultation with the patient. |
| **6005** | Local status | Local status |
| **6006** | Differential diagnosis consultation | This includes a comprehensive examination that assesses: (i) neurological status, (ii) orthopaedic status, (iii) chiropractic status, and (iv) any other special assessments required to establish a differential diagnosis.  Note: 6006 cannot be cumulated with 6001 or 6002 and can only be used once per “case” or “relapse”. A “case” is defined as the evaluation and treatment of one and the same symptom complex presented at the time of the initial consultation (Chiropractor tariff UV/IV/MV from 1.12.2017).  A “relapse” is a flare-up of an accident/illness that had supposedly been cured. It results in medical treatment, possibly even incapacity to work (Decision of the Federal Supreme Court: BGE 105 V31 of 13.3.1979). |
| **6007** | Additional time for figure 6002, per 15 min | If a further consultation (item 6002) exceptionally lasts longer than 15 minutes, a surcharge will be made for each full or commenced quarter of an hour. This refers exclusively to the duration of the consultation, not to the duration of the treatment. |
| **6009** | File study, per 5 min |  |
| **6021** | Registration for UVG and MV patients |  |
| **6022** | Simple report (formalized report) |  |
| **6023** | Detailed report (free text) |  |
| **6024** | Referral or final report |  |
| **6025** | Doctor's report IV |  |
| **6026** | Medical interim report IV |  |
| **04** | Imaging techniques | Explanations to chapter 4ff  The fees according to chapters 4ff are generally added to the consultation fee. They are to be charged alone if no measures covered by the consultation fee are provided.  The fees include a radiological report and a copy of the image. The images must be technically and setting-wise flawless and must be in the required format. All films must be labeled in such a way that confusion is impossible (name, first name, year of birth, page designation and date of recording). The tariff distinguishes between first and further recordings. Further exposures are those taken in the same session or on the same day of the same part of the body in different positions, beam direction and hardness. A comparative or control image taken at a later session or on a subsequent day is to be charged as a first image only if its postponement to later is indicated for medical reasons. The responsibility for technical quality assurance (QA) lies with the Federal Office of Public Health (FOPH). The other QA measures are defined in a separate contract between SCG and the insurers in accordance with Article 5 of the tariff agreement. Images of body regions taken externally by the chiropractor for diagnostic purposes and obtained by means of imaging procedures will be remunerated in accordance with the applicable tariff. |
| **04.0**  **1** | Spinal regions (cervical spine, thoracic spine, lumbar spine, pelvis, thorax) |  |
| **6031** | first exposure (per spinal region) |  |
| **6032** | Each additional exposure (per spinal region) |  |
| **04.0**  **2** | Extremities: |  |
| **6041** | First exposure (per limb region) |  |
| **6042** | Each additional exposure (per limb region) |  |
| **05** | Chiropractic therapy | This includes, individually or in combination, the therapeutic competencies encompassed by the chiropractor's scope of training, examination, and professional license, specifically:  - Manipulation and joint mobilization  - manual stretching, connective tissue treatment, manual reflex therapy  - Physical-therapeutic measures that serve to support the specific chiropractic intervention.  Items 6051 and 6052 as well as 6052 and 6053 cannot be used cumulatively. |
| **6051** | Chiropractic treatment (1-3 treatment regions) |  |
| **6052** | Complex, chiropractic treatment (more than 3 treatment regions) | Treatment regions according to 6051 and 6052 are:  1. cranium/ cervical spine  2. thoracic spine/ rib thorax  3. lumbar spine  4. pelvis  5. extremities |
| **6053** | Neuromuscular reflex therapy |  |
| **06** | Physical therapy, counseling, and muscular rehabilitation |  |
| **6056** | passive, physical-therapeutic measures | (max. twice per consultation) |
| **6057** | active, remedial gymnastic measures, muscular rehabilitation | (max. once per consultation) |
| **07** | Support, fixation, and other permanent bandages | The application of prefabricated (removable or fixed) permanent dressings is included in the consultation fee.  The material is charged at cost price (cost price + 15%). |
| **6071** | Application of adhesive and support bandages made of hardening material | Supporting bandages made of hardening material, adhesive bandages |
| ^a^See Central Office for Medical Tariffs (Zentralstelle für Medizinaltarife, ZMT) for a thorough description of tariff codes: [**https://www.mtk-ctm.ch/de/**](https://www.mtk-ctm.ch/de/).  ^b^Adapted from German.  **Any chiropractic care—operational definition:** Any chiropractic care operationalised as service tariff type 324 (chiropractic care), with billing codes: 6001, 6002, 6003, 6004, 6005, 6006, 6007, 6008, 6009, 6011, 6021, 6022, 6023, 6024, 6025, 6031, 6031, 6032, 6041, 6042, 6051, 6052, 6053, 6054, 6056, 6057, 6071.  **Only chiropractic care prevalence—operational definition:** Only chiropractic care prevalence operationalised as only occurrence of non-chiropractic-care-incidence codes (6002, 6003, 6004, 6005, 6007, 6008, 6009, 6011, 6021, 6022, 6023, 6024, 6025, 6031, 6032, 6041, 6042, 6051, 6052, 6053, 6054, 6056, 6057, 6071) in index year (i.e., does not include codes 6001 or 6006). | | |

# **sTable 3.** Pharmaceutical cost groups (PCG)

| **Code** | **Diagnosis** | **Comorbidity Group** |
| --- | --- | --- |
| ABH | Addiction without nicotine | Psychiatric diseases |
| ADH | ADHD | Psychiatric diseases |
| AIK | Autoimmune disorders | Autoimmune diseases |
| ALZ | Alzheimer | Neurological diseases |
| AST | Asthma | Pulmonic diseases |
| BSR | Bipolar disorder | Psychiatric diseases |
| CAR | Cardiac disease | Cardiovascular diseases |
| COP | COPD / severe asthma | Pulmonic diseases |
| DEP | Depression | Psychiatric diseases |
| DM1 | Type I Diabetes | Endocrine diseases |
| DM2 | Type II Diabetes | Endocrine diseases |
| DMH | Diabetes with hypertension | Endocrine diseases |
| EPI | Epilepsy | Neurological diseases |
| GLA | Glaucoma | Other |
| HCH | High Cholesterol | Cardiovascular diseases |
| HIV | HIV / AIDS | Other |
| KHO | Hormone-sensitive Cancers | Cancer |
| KRE | Cancer | Cancer |
| KRK | Cancer complex | Cancer |
| MCR | Crohn's disease/ Ulcerative colitis | Autoimmune diseases |
| MSK | Multiple Sclerosis | Neurological diseases |
| NIE | Kidney diseases | Other |
| PAH | Hypertension | Cardiovascular diseases |
| PAR | Parkinson's disease | Neurological diseases |
| PSO | Psoriasis | Autoimmune diseases |
| PSY | Psychosis | Psychiatric diseases |
| RHE | Rheumatism | Other |
| SMC | Chronic pain without opioid use | Pain syndromes |
| SMN | Neuropathic pain | Pain syndromes |
| THY | Thyroid diseases | Endocrine diseases |
| TRA | Transplantation | Other |
| WAS | Growth disturbance | Other |
| ZFP | Cystic fibrosis / Pancreatic enzymes | Pulmonic diseases |
| ZNS | Central nerve system diseases excluding Multiple Sclerosis | Neurological diseases |

# **sTable 4.** Characteristics of incidence cases in both 2018 and 2019

|  | **Incidence 2018 and 2019**  **N = 4,749** | **Incidence and ≤ 180 days^a^**  **N = 473** | **Incidence and ≤ 90 days^b^**  **N = 99** |
| --- | --- | --- | --- |
| Sex – N (%) |  |  |  |
| Women | 2,725 (57.4) | 265 (56.0) | 51 (51.5) |
| Men | 2,024 (42.6) | 208 (44.0) | 48 (48.5) |
| Missing | 0 (0) | 0 (0) | 0 (0) |
| Age (years) – mean (SD) | 49.6 (17.7) | 49.0 (17.5) | 48.8 (17.1) |
| Age group – N (%) |  |  |  |
| 0 to 17 years | 212 (4.5) | 22 (4.7) | 3 (3.0) |
| 18 to 29 years | 439 (9.2) | 49 (10.4) | 9 (9.1) |
| 30 to 69 years | 3,411 (71.8) | 341 (72.1) | 74 (74.7) |
| 70 years or older | 687 (14.5) | 61 (12.9) | 13 (13.1) |
| Missing | 0 (0) | 0 (0) | 0 (0) |
| Region – N (%) |  |  |  |
| Lake Geneva region | 837 (17.6) | 105 (22.2) | 36 (36.4) |
| Espace Mittelland | 1,272 (26.8) | 102 (21.6) | 10 (10.1) |
| Northwestern Switzerland | 459 (9.7) | 48 (10.1) | 6 (6.1) |
| Zurich | 1,306 (27.5) | 148 (31.3) | 34 (34.3) |
| Eastern Switzerland | 469 (9.9) | 29 (6.1) | 3 (3.0) |
| Central Switzerland | 360 (7.6) | 34 (7.2) | 7 (7.1) |
| Ticino | 46 (1.0) | 7 (1.5) | 3 (3.0) |
| Missing | 0 (0) | 0 (0) | 0 (0) |
| Deceased – N (%) |  |  |  |
| Deceased year – N (%) |  |  |  |
| 2017 | 0 (0) | 0 (0) | 0 (0) |
| 2018 | 0 (0) | 0 (0) | 0 (0) |
| 2019 | 0 (0) | 0 (0) | 0 (0) |
| 2020 | 17 (0.4) | 3 (0.6) | 2 (2.0) |
| 2021 | 27 (0.6) | 2 (0.4) | 2 (2.0) |
| Area of residence by language – N (%) |  |  |  |
| German | 3,716 (78.2) | 350 (74.0) | 61 (61.6) |
| French | 981 (20.7) | 116 (24.5) | 35 (35.4) |
| Italian | 47 (1.0) | 7 (1.5) | 3 (3.0) |
| Romansch | 5 (0.1) | 0 (0) | 0 (0) |
| Missing | 0 (0) | 0 (0) | 0 (0) |
| Health insurance model – N (%) |  |  |  |
| Standard (free choice) model | 2,555 (53.8) | 246 (52.0) | 56 (56.6) |
| Family physician model (binding) | 1,150 (24.2) | 125 (26.4) | 27 (27.3) |
| Telemedicine model (binding) | 598 (12.6) | 62 (13.1) | 9 (9.1) |
| Telemedicine model (nonbinding) | 446 (9.4) | 40 (8.5) | 7 (7.1) |
| Missing | 0 (0) | 0 (0) | 0 (0) |
| Health insurance deductible – N (%) |  |  |  |
| Level 1, ≤500 CHF | 2,911 (61.3) | 292 (61.7) | 54 (54.5) |
| Level 2, 501–1500 CHF | 1,117 (23.5) | 110 (23.3) | 25 (25.3) |
| Level 3, 1501–2500 CHF | 721 (15.2) | 71 (15.0) | 20 (20.2) |
| Missing | 0 (0) | 0 (0) | 0 (0) |
| Comorbidities in index year – N (%) |  |  |  |
| None | 3,471 (73.1) | 332 (70.2) | 70 (70.7) |
| One | 962 (20.3) | 118 (24.9) | 24 (24.2) |
| Two | 244 (5.1) | 19 (4.0) | 4 (4.0) |
| Three or more | 72 (1.5) | 4 (0.8) | 1 (1.0) |
| In-hospital index visit – N (%) | 79 (1.7) | 19 (4.0) | 11 (11.1) |
| ^a^ ≤ 180 days between 2018 and 2019 chiropractic index visits.  ^b^ ≤ 90 days between 2018 and 2019 chiropractic index visits. | | | |

# **sTable 5.** Crude incidence of chiropractic care and number of chiropractors (supply) in 2018 and 2019

| **Region** | **Incidence 2018**  **(95% CI)** | **General 2018^a^** | **Number of chiropractors 2018^b^** | **Density per 100,000—2018** | **Incidence 2019 (95%CI)** | **General 2019^a^** | **Number of chiropractors 2019^b^** | **Density per 100,000—2019** |
| --- | --- | --- | --- | --- | --- | --- | --- | --- |
| **Lake Geneva** | 1,248 (1,297 to 1,401) | 1,642,580 | 61 | 3.7 | 1,400 (1349 to 1451) | 1,654,751 | 62 | 3.7 |
| **Espace Mittelland** | 1,895 (1,840 to 1,951) | 1,877,154 | 86 | 4.6 | 1,857 (1,804 to 1,911) | 1,886,584 | 88 | 4.7 |
| **Northwestern Switzerland** | 1,119 (1,069 to 1,171) | 1,161,105 | 26 | 2.2 | 1,102 (1,054 to 1,152) | 1,171,157 | 27 | 2.3 |
| **Zurich** | 1,734 (1,686 to 1,784) | 1,520,968 | 50 | 3.3 | 1,839 (1,790 to 1,889) | 1,539,275 | 58 | 3.8 |
| **Eastern Switzerland** | 1,469 (1,404 to 1,536) | 1,176,321 | 33 | 2.8 | 1,387 (1,326 to 1,451) | 1,183,813 | 31 | 2.6 |
| **Central Switzerland** | 1,657 (1,575 to 1,744) | 813,056 | 29 | 3.6 | 1,708 (1,625 to 1,795) | 818,962 | 27 | 3.3 |
| **Ticino** | 531 (479 to 589) | 353,343 | 12 | 3.4 | 540 (488 to 597) | 351,491 | 12 | 3.4 |

^a^ General (permanent resident) population of Switzerland [16,17]; Reference day: 31 December; Data source: Population and Households Statistics STATPOP.

^b^ Number of chiropractors includes chiropractic residents; Two chiropractors in Lichtenstein were excluded.

# **sTable 6.** Characteristics of the general population of Switzerland and the Helsana source population

|  | **General 2018^a^**  **N =** **8,544,527** | **Source 2018**  **N = 1,137,904** | **General 2019^a^**  **N = 8,606,033** |  | **Source 2019**  **N = 1,196,760** |  |  |
| --- | --- | --- | --- | --- | --- | --- | --- |
| Sex – N (%) |  |  |  |  |  |  |  |
| Women | 4,307,406 (50.4) | 588,130 (51.7) | 4,337,170 (50.4) |  | 616,446 (51.5) |  |  |
| Men | 4,237,121 (49.6) | 549,774 (48.3) | 4,268,863 (49.6) |  | 580,314 (48.5) |  |  |
| Age group – N (%) |  |  |  |  |  |  |  |
| 0 to 17 years | 1,530,231 (17.9) | 204,070 (17.9) | 1,542,361 (17.9) |  | 219,993 (18.4) |  |  |
| 18 to 29 years | 1,231,960 (14.4) | 160,803 (14.1) | 1,220,187 (14.2) |  | 168,110 (14.0) |  |  |
| 30 to 69 years | 4,633,031 (54.2) | 567,167 (49.8) | 4,668,297 (54.2) |  | 600,472 (50.2) |  |  |
| 70 years or older | 1,149,305 (13.5) | 205,864 (18.1) | 1,175,188 (13.7) |  | 208,185 (17.4) |  |  |
| Region – N (%) |  |  |  |  |  |  |  |
| Lake Geneva region | 1,642,580 (19.2) | 188,383 (16.6) | 1,654,751 (19.2) |  | 201,709 (16.9) |  |  |
| Espace Mittelland | 1,877,154 (22.0) | 228,634 (20.1) | 1,886,584 (21.9) |  | 243,284 (20.3) |  |  |
| Northwestern Switzerland | 1,161,105 (13.6) | 162,307 (14.3) | 1,171,157 (13.6) |  | 173,953 (14.5) |  |  |
| Zurich | 1,520,968 (17.8) | 273,414 (24.0) | 1,539,275 (17.9) |  | 285,189 (23.8) |  |  |
| Eastern Switzerland | 1,176,321 (13.8) | 128,973 (11.3) | 1,183,813 (13.8) |  | 133,821 (11.2) |  |  |
| Central Switzerland | 813,056 (9.5) | 88,025 (7.7) | 818,962 (9.5) |  | 89,361 (7.5) |  |  |
| Ticino | 353,343 (4.1) | 68,147 (6.0) | 351,491 (4.1) |  | 69,434 (5.8) |  |  |
| Missing | - | 21 (0) | - |  | 9 (0) |  |  |
| ^a^ General (permanent resident) population of Switzerland [16,17]; Reference day: 31 December; Data source: Population and Households Statistics STATPOP.  ^b^ Combines binding and nonbinding insurance models. | | | | | | |  |
